# Association of *CD14* rs2569190 polymorphism with mortality in shock septic patients who underwent major cardiac or abdominal surgery: A retrospective study

**DOI:** 10.1038/s41598-018-20766-7

**Published:** 2018-02-09

**Authors:** María Ángeles Jiménez-Sousa, Pilar Liu, Luz María Medrano, Amanda Fernández-Rodríguez, Raquel Almansa, Esther Gómez-Sánchez, Lucía Rico, Mario Lorenzo, Alejandra Fadrique, Eduardo Tamayo, Salvador Resino

**Affiliations:** 1 Unidad de Infección Viral e Inmunidad. Centro Nacional de Microbiología, Instituto de Salud Carlos III, Majadahonda, Spain; 2 Departamento de Anestesiología y Reanimación, Hospital Clínico Universitario de Valladolid, Valladolid, Spain; 3 Unidad de Investigación Médica en Infección e Inmunidad, Hospital Clínico Universitario de Valladolid-IECSCYL, Valladolid, Spain, On behalf of the Group of Biomedical Research in Critical Care Medicine (BioCritic), Valladolid, Spain

## Abstract

The aim of this study was to investigate the relationship between the *CD14* rs2569190 polymorphism and death related to septic shock in white European patients who underwent major cardiac or abdominal surgery. We carried out a retrospective study in 205 septic shock patients. The septic shock diagnosis was established by international consensus definitions. The outcome variable was the death within 28, 60 and 90 days after septic shock diagnosis. The *CD14* rs2569190 polymorphism was analyzed by Agena Bioscience’s MassARRAY platform. For the genetic association analysis with survival was selected a recessive inheritance model (GG vs. AA/AG). One hundred thirteen out of 205 patients (55.1%) died with a survival median of 39 days (95%CI = 30.6; 47.4). Patients with rs2569190 GG genotype had shorter survival probability than rs2569190 AA/AG genotype at 60 days (62.3% vs 50%; p = 0.035), and 90 days (62.3% vs 52.6%; p = 0.046). The rs2569190 GG genotype was associated with increased risk of septic shock-related death in the first 60 days (adjusted hazard ratio (aHR) = 1.67; p = 0.016) and 90 days (aHR = 1.64; p = 0.020) compared to rs2569190 AA/AG genotype. In conclusion, the presence of *CD14* rs2569190 GG genotype was associated with death in shock septic patients who underwent major surgery. Further studies with bigger sample size are required to verify this relationship.

## Introduction

Sepsis is a major cause of death from infection, although mortality from sepsis has decreased due to improved supportive care and evidence-based guidelines for diagnosis and timely intervention^1,2^. Besides, septic shock kills a large number of patients in hospitals, mainly in intensive care unit (ICU)^1–3^. In general terms, sepsis is a life-threatening organ dysfunction caused by altered host response to infection; and septic shock is a subset of sepsis with circulatory and cellular/metabolic abnormalities, which has been related to higher risk of dying^4^.

In many cases, the stabilization of the clinical condition in septic patients is reached by the use of anti-infective treatments together to aggressive organ failure supports^1^. Nevertheless, these patients are susceptible to ICU-related complications, which have a notable repercussion on their early and late prognosis^5,6^. Thus, multiple organ failure caused by the primary infection could mainly explain early deaths and ICU-related complications such as nosocomial infections and mesenteric ischemia could lead to late deaths^5^. The dysfunction at the molecular level is reflected significantly by the outcome of these patients. In sepsis, the vascular endothelium is considerably affected, appearing changes in the mitochondrial functionality because of the repeated hypoxia episodes. This mitochondrial damage may induce a series of cellular and pathophysiological dysfunctions, increasing the chances of multiple organ dysfunction syndrome (MODS) and death^7^.

Lipopolysaccharide (LPS) or endotoxin plays a major role in initiating the typical septic inflammatory cascade with overproduction of proinflammatory cytokines^8^. LPS is the major component of the outer membrane of gram-negative bacteria, but LPS may also trigger inflammation in gram-positive and fungal sepsis due to the release of excessive amounts of gut-derived LPS during intestinal hypoperfusion^9,10^.

CD14, together with toll-like receptor 4 (TLR4) and lymphocyte antigen 96 (LY96, also called MD-2), may bind LPS resulting in NF-kB activation and the production of proinflammatory cytokine^11^. In this regard, there are a high number of genes, which are induced by NF-kB, that are implicated in the cellular response against infection and promote the synthesis of pro-inflammatory mediators. Additionally, several microRNAs have been involved in the NF-kB activity and modulation of the immune response, resulting in a worsening of the sepsis with developing of MODS and death. Additionally, several microRNAs have been involved in the NF-kB activity and modulation of the immune response, resulting in a worsening of the sepsis with developing of MODS and death^12^.

CD14, a 53–55 kDa glycoprotein, is expressed on the surface (mCD14) of myeloid cells (monocytes, macrophages, and neutrophils) and non-myeloid cells (endothelial and epithelial cells)^11^. CD14 may also be found in a soluble form (sCD14), which apparently derives from the secretion of CD14 or from proteolysis of mCD14^11^. Both mCD14 and sCD14 are critical for LPS-dependent signal transduction. Besides, CD14 may also participate in immune cell activation by gram-positive cell-wall components^13–15^. The role of CD14 and the inflammatory response are essential to eradicate primary infections and prevent the acquisition of secondary infections in patients with sepsis^16^. However, CD14 also has a pivotal role in initiating and perpetuating the pro-inflammatory response during the course of sepsis^17^.

A single nucleotide polymorphism (SNP) detected at position −159 in the promoter region of the *CD14* gene (rs2569190) has been related to Sp protein. The presence of a T at −159 decreases the homology between the *CD14* promoter GC box and the Sp consensus sequence.^18^, leading to subsequent increases in CD14 production^18,19^. Thus, the rs2569190 SNP increases the level of pro-inflammatory response following LPS stimulation because it could modulate the transcriptional activity of CD14^19,20^. The *CD14* rs2569190 polymorphism has been related to susceptibility to sepsis, as well as the outcome of sepsis^17^, but there are controversial results due to the small sample size and the heterogeneity of the patients included in the previous studies.

In this study, we analyzed the relationship between the *CD14* rs2569190 polymorphism and death in European septic shock patients who underwent major surgery.

## Results

### Population characteristics

Epidemiological and clinical characteristics of septic shock patients at the moment of septic shock diagnosis and stratified by *CD14* rs2569190 genotype are shown in Table 1. In brief, 63.9% were male, the median age was 73 years and 54.6% and 44.9% of patients had hypertension and heart disease, respectively. Regarding the surgery, 40% of patients were underwent cardiac surgery and 63.4% of surgery were urgent. The most predominant pathogens were gram-negatives (52.7%). Regarding site of infection, 46.8% and 47.3% of patients had peritonitis or pneumonia, respectively. When the population was divided taken into consideration *CD14* rs2569190 genotype under a recessive genetic model, we did not find any significant differences between groups.

**Table 1:** Summary of epidemiological and clinical characteristics of septic shock patients who underwent major surgery according to *CD14* polymorphism.

Characteristics	All Patients	*CD14* rs2569190 polymorphism		
		AA/AG	GG	p-value(^*^)
No. patients	205	152	53	—
Gender (male)	131 (63.9%)	100 (65.8%)	31 (58.5%)	0.341
Age (years)	73. (63; 79.5)	72 (63; 79.7)	77 (67.5; 79.5)	0.137
Prior or pre-existing conditions				
Smoker	36 (17.6%)	27 (17.8%)	9 (17.0%)	0.897
Alcoholism	15 (7.3%)	9 (5.9%)	6 (11.3%)	0.194
Obesity	30 (14.6%)	18 (11.8%)	12 (22.6%)	0.055
Diabetes	26 (12.7%)	17 (11.2%)	9 (17.0%)	0.275
Heart disease	92 (44.9%)	71 (46.7%)	21 (39.6%)	0.372
Chronic obstructive pulmonary disease	36 (17.6%)	24 (15.8%)	12 (22.6%)	0.259
Hypertension	112 (54.6%)	81 (53.3%)	31 (58.5%)	0.513
Chronic kidney disease	30 (14.6%)	23 (15.1%)	7 (13.2%)	0.733
Cancer	48 (23.4%)	31 (20.4%)	17 (32.1%)	0.084
Liver disease	9 (4.4%)	6 (3.9%)	3 (5.7%)	0.600
Surgery				
Cardiac (versus abdominal)	82 (40.0%)	62 (40.8%)	20 (37.7%)	0.696
Emergency (versus scheduled)	130 (63.4%)	97 (63.8%)	33 (62.3%)	0.840
Severity indexes				
SOFA score	9 (7; 10.5)	9 (7; 10)	9 (7; 11)	0.510
APACHE II score	16 (14; 19)	16 (13; 19)	17 (14; 21.5)	0.060
Infection				
Gram-positive	102 (49.8%)	81 (53.3%)	21 (39.6%)	0.087
Gram-negative	108 (52.7%)	78 (51.3%)	30 (56.6%)	0.507
Fungus	41 (20.0%)	34 (22.4%)	7 (13.2%)	0.151
Catheter bacteraemia	70 (34.1%)	55 (36.2%)	15 (28.3%)	0.297
Surgical site infection	49 (23.9%)	35 (23.0%)	14 (26.4%)	0.618
Urinary tract infection	24 (11.7%)	15 (9.9%)	9 (17.0%)	0.165
Endocarditis	10 (4.9%)	8 (5.3%)	2 (3.8%)	0.665
Peritonitis	96 (46.8%)	72 (47.4%)	24 (45.3%)	0.793
Pneumonia	97 (47.3%)	69 (45.4%)	28 (52.8%)	0.351

Values are expressed as absolute count (percentage) and median (percentile 25; percentile 75).

(^*^)P-values were calculated by Chi-square test or Fisher’s exact test for categorical variables and Mann-Whitney test for continuous variables. Statistically significant differences are shown in bold.

Note that patients may have had more than one organism cultured.

Abbreviations: SOFA, sequential organ failure assessment; APACHE, acute physiology and chronic health evaluation; CD14, cluster of differentiation 14.

### Frequency of the *CD14* polymorphism

The frequencies of *CD14* rs2569190 polymorphism in patients and control groups (patients with systemic inflammatory response syndrome (SIRS) and healthy subjects from the Iberian populations in Spain (IBS)^21^) are shown in Table 2. The *CD14* rs2569190 polymorphism was in Hardy–Weinberg equilibrium (HWE) (p > 0.05) and showed missing values <5%. Significant differences in allelic and genotypic frequencies were not found among groups.

**Table 2:** Frequencies of alleles and genotypes for *CD14* rs2569190 polymorphism in septic shock patients compared to Iberian populations in Spain from 1000 Genomes Project data (http://www.1000genomes.org/1000-genomes-browsers) and SIRS patients.

	SNP	IBS population	SIRS patients	Septic shock patients	p-value^(a)^	p-value^(b)^
No.		107	259	205		
HWE (p-value)		0.149	0.768	0.238		
Alleles	G	51.4%	51.2%	48.8%	0.939	0.660
	A	48.6%	48.8%	51.2%	—	—
Genotypes	AA	29.9%	24.1%	28.5%	0.235	0.765
	AG	43.0%	49.4%	45.9%	0.294	0.629
	GG	27.1%	26.5%	25.9%	0.988	0.812

P-values were calculated by Chi-squared test: (^a^) differences between IBS population and septic shock patients; (^b^) differences between SIRS patients and septic shock patients.

Abbreviations: SIRS, patients with systemic inflammatory response syndrome; IBS, Iberian populations in Spain; HWE, Hardy-Weinberg equilibrium; CD14, cluster of differentiation 14.

### *CD14* polymorphism and septic shock-related death

**Figure 1:**
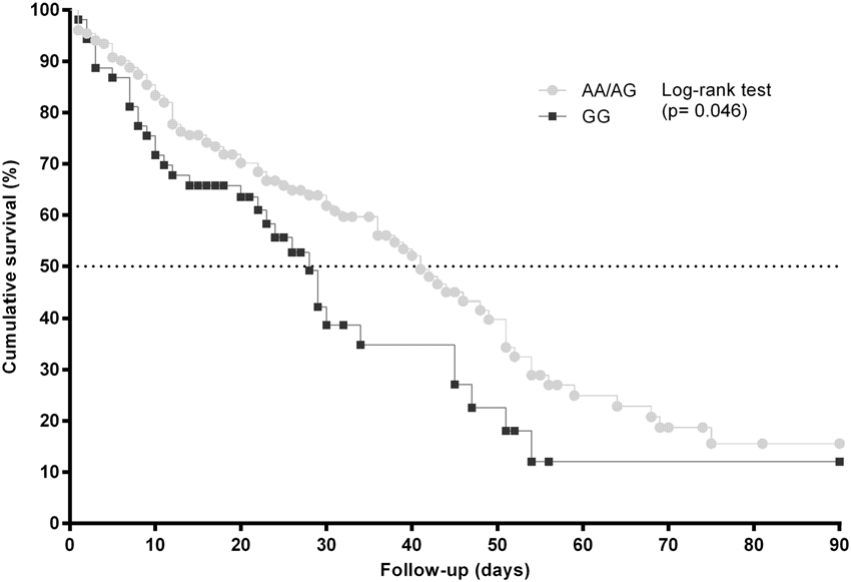
Survival analysis (Kaplan-Meier curve) regarding to *CD14* rs2569190 polymorphism in septic shock patients who underwent major cardiac or abdominal surgery. P-values were calculated by log-rank test (Kaplan-Meier curve).

For the genetic association study, a recessive inheritance model (GG vs. AA/AG) was selected, due to it was the model that best fit to our data. The survival probabilities at different time points (28, 60, and 90 days) after septic shock diagnosis are shown in Table 3. One hundred thirteen out of 205 patients (55.1%) died with a survival median of 39 days (95%CI = 30.6; 47.4). Patients with *CD14* rs2569190 GG genotype had higher death probability than *CD14* rs2569190 AA/AG genotype at 60 days (p = 0.035), and 90 days (p = 0.046) (Fig. 1). The death risks at 28, 60, and 90 days obtained by Cox regression for the *CD14* rs2569190 polymorphism are shown in Table 4. The *CD14* rs2569190 GG genotype was related to bigger adjusted risk of death related to septic shock at day 60 (adjusted hazard ratio (aHR) = 1.67; p = 0.016) and day 90 (aHR = 1.64; p = 0.020) than *CD14* rs2569190 AA/AG genotype.

**Table 3:** Survival probabilities at 28, 60, and 90 days (Kaplan-Meier product limit method) for *CD14* rs2569190 polymorphism in septic shock patients who underwent major cardiac or abdominal surgery.

Points censoring	All Patients	*CD14* rs2569190 polymorphism		
		rs2569190 AA/AG	rs2569190 GG	*P*-value (log-rank test)
28 days	36.1%	32.9%	45.3%	0.095
60 days	53.2%	50.0%	62.3%	0.035
90 days	55.1%	52.6%	62.3%	0.046

Abbreviations: CD14, cluster of differentiation 14; p-value, level of significance.

**Table 4:** Risk of death regarding *CD14* rs2569190 polymorphism in septic shock patients who underwent major cardiac or abdominal surgery.

	Univariate			Multivariate		
	HR	95%CI	p-value	aHR	95%CI	p-value(^*^)
**The first 28 days**						
rs2569190 GG	1.50	0.92; 2.45	0.101	1.55	0.95; 2.53	0.077
Emergency surgery				1.86	1.00; 3.46	0.049
APACHE-II score				1.04	1.00; 1.08	0.067
Peritonitis				2.28	1.35; 3.85	0.002
Heart disease				1.97	1.20; 3.22	0.007
**The first 60 days**						
rs2569190 GG	1.54	1.02; 2.33	0.039	1.67	1.10; 2.54	0.016
Emergency surgery				1.86	1.18; 2.92	0.007
Peritonitis				1.84	1.19; 2.86	0.006
Heart disease				1.72	1.13; 2.63	0.012
Liver disease				1.91	0.82; 4.47	0.133
**The first 90 days**						
rs2569190 GG	1.50	1.00; 2.26	0.050	1.64	1.08; 2.48	0.020
Emergency surgery				1.89	1.21; 2.95	0.005
Peritonitis				1.83	1.18; 2.82	0.007
Heart disease				1.70	1.12; 2.58	0.012
Liver disease				1.92	0.82; 4.47	0.132

(^*^)P-values were calculated by Cox regression adjusting for the most important clinical and epidemiological characteristics (see statistical analysis section).

Abbreviations: CD14, cluster of differentiation 14; HR, hazard ratio; aHR, adjusted hazard ratio; 95%CI, 95% confidence interval; p-value, level of significance.

Finally, the risk of death regarding *CD14* rs2569190 polymorphism was separately assessed for patients underwent cardiac and abdominal surgery. In patients underwent cardiac surgery, the *CD14* rs2569190 GG carriers had higher risk of death related to septic shock during the first 60 days [aHR = 2.26 (95%CI = 1.08; 4.82); p = 0.029] and 90 days [aHR = 2.07 (95%CI = 1.01; 4.29); p = 0.049] than *CD14* rs2569190 AA/AG carrier; whereas *CD14* rs2569190 GG carriers who underwent abdominal surgery did not have any significant association with mortality related to septic shock during the first 28, 60 and 90 days after diagnosis.

## Discussion

The genetic variation at genes of innate immunity may have influence on the occurrence of sepsis and death^22,23^. In this study, we found a relationship between *CD14* rs2569190 polymorphism and mortality in European septic shock patients who underwent major surgery. Specifically, patients with *CD14* rs2569190 GG genotype had increased risk of death related to septic shock, suggesting that *CD14* rs2569190 polymorphism may have an important function in the pathogenesis of septic shock. To our knowledge, this is the first report describing these findings in patients who underwent major surgery, while remaining consistent with observations in critically ill patients with medical conditions^24,25^ or burn injury^26,27^.

To date, discrepant results have been described for the association between *CD14* rs2569190 polymorphism and sepsis and death^17^.

In 2013, a meta-analysis of Zhang *et al*.^17^ suggested that the *CD14* rs2569190 polymorphism could not be a relevant risk factor for sepsis and mortality because only weak associations were found in Asian populations and septic shock patients. They found that patients with AA/AG genotypes had a tendency towards higher risk of death than patients with GG genotype^17^. However, our results are not consistent with this meta-analysis, since we observed a beneficial effect of the AA/AG genotype on septic shock survival in European people. Note that the meta-analysis by Zhang *et al*. did not provide information about the association of *CD14* rs2569190 polymorphism with septic-shock survival in European populations. This was probably due to the low number of sepsis mortality-related studies included in the meta-analysis, which precluded the stratification by ethnicity^17^. In addition, other specific studies have associated *CD14* rs2569190 G allele with higher risk of sepsis and death in people of European origin, which may support our results^18,26–30^.

The A to G polymorphism at position-159 in the promoter region of the *CD14* gene (rs2569190) could cause differential activity in promoter constructs. In this context, rs2569190 G allele seems to be implicated in down-regulation of *CD14* transcription and lower expression of mCD14 and circulating levels of sCD14^19,20^. With this in mind, previous reports suggest that the biological correlation of the *CD14* rs2569190 polymorphism with the survival in patients with A-allele could be attributed to the strongest pro-inflammatory response among patients, consequence of higher CD14 expression^19,20,31–33^. This assumption is consistent with the increasingly prevailing opinion that the major problem of sepsis patients is the predominant state of immunosuppression characterized by a reduced pro-inflammatory status and increased anti-inflammatory response^16^. In our study, assays for determination of mCD14 or sCD14 could support the effect observed for *CD14* rs2569190 polymorphism, however CD14 values were not available in our study.

Additionally, sCD14 level has been recently described as a novel marker for postoperative mortality in elective cardiac surgery patients, showing implication at different times (30-day, 6-month and 2 year-mortality)^34^. In our study, we observed an effect of *CD14* rs2569190 polymorphism at 60 and 90-day mortality. As the mortality is a cumulative variable, a more limited number of patients suffering death at 28-day mortality compared to 60- and 90-day mortality was found, which may have decreased the statistical power of our analysis. However, we found a trend of association in the multivariate model (p = 0.077), which would be in concordance with previous findings described by Mansur *et al*., who found that *CD14* rs2569190 may act as a prognostic variable for the short-term outcome due to its association with 30-day mortality in patients with sepsis^29^.

In this study, we were not able to carry out a study to decipher the mechanisms underlying this association because we did not have access to other samples of these patients. However, we carried out an *in silico* study for assessing the regulatory features of this polymorphism taking into account chromatin state of the region surrounding variant, regulatory elements overlapped with variant, and variant’s potential target genes. We used rVarBase database^34,35^, which uses experimentally supported regulatory elements from ENCODE and other data resources to make relevant annotation. We found that rs2569190 polymorphism, located within the promoter of *CD14*, influence the chromatin states in different cell lines such as neutrophils, monocytes, natural killer cells, T cells and B cells from peripheral blood as well as hepatic cells from the liver. Keeping this in mind, we stress the importance of further investigations to study the mechanisms that could be influencing the association of *CD14* rs2569190 with septic shock-related death.

We compared frequencies of *CD14* rs2569190 alleles and genotypes between septic shock and SIRS patients recruited in this study and healthy people obtained from IBS^35^. No significant differences among groups were observed, indicating no bias for the distribution of *CD14* rs2569190 polymorphism in our study. In addition, a particular strength of this study lies in the fact that there not were baseline differences in clinical and epidemiological variables according to *CD14* rs2569190 genotypes (AA/AG vs GG). Despite this, we included the clinical and epidemiological variables into the multivariate Cox-regression analysis, and *CD14* rs2569190 GG genotype remained a significant risk factor for mortality in 60 days and 90 days. In addition to the *CD14* rs2569190 polymorphism, we observed several factors that were related to death such as emergency surgery, APACHE-II score, peritonitis, and heart disease. However, although we evaluated an elevated number of known variables influencing sepsis, other factors not included in this study could have an important role in this issue. Besides, other genetic variants could synergistically act with *CD14* rs2569190 polymorphism, participating in the occurrence of death in septic patients. These factors could be other previously described SNPs within the inflammatory-related genes that have been related to death^36^.

In addition, note that *CD14* rs2569190 polymorphism could also influence other sepsis types. In fact, we also investigated the association between *CD14* rs2569190 polymorphism and severe sepsis by using a small cohort of 43 patients who developed severe sepsis after cardiac or abdominal surgery (data not shown), finding an association between *CD14* rs2569190 polymorphism and 90-day mortality (p = 0.033). This underlines the probable role of *CD14* rs2569190 polymorphism on the other sepsis types, but further analysis with bigger sample size would be needed to corroborate such association.

Finally, it should be stressed that this retrospective study has a relatively small sample size, which could increase the number of false positives or negatives. Complex human diseases, such as sepsis, are under the control of many genes that contribute with modest individual effects. The sample size could be an important issue in this regard, since only big effects would be detected in small populations. However, our cohort was homogenous because we only included septic shock patients, without including other stages of sepsis. We also carried out a follow-up study and survival analysis, which are often much more sensitive to detect statistical associations in cohort studies. Besides, the fact of finding a significant association at different times even when *CD14* rs2569190 polymorphism is studied in combination with other important prognostic factors, reinforces the role of this polymorphism on septic shock-related death. However, further studies with bigger sample size would be needed to corroborate such association. Moreover, in our study, the association of *CD14* rs2569190 polymorphism with mortality related to septic shock was only observed in patients underwent major cardiac surgery; whereas patients underwent abdominal surgery did not show any significant association. In this setting, note that the reduced sample size of surgery groups could influence the different pattern observed between cardiac and abdominal surgery. Besides, the association should also be investigated in different racial groups. Finally, other *CD14* polymorphisms could provide interesting information and thus, they should also be evaluated in further studies.

In conclusion, *CD14* rs2569190 GG genotype was associated with mortality in patients with septic shock who underwent major surgery. The *CD14* rs2569190 polymorphism could allow an improved management of death risk in septic shock patients. Further studies with bigger sample size are required to verify this relationship.

## Materials and Methods

### Patients and study design

We performed a retrospective study in 205 patients who developed septic shock after major cardiac or abdominal surgery. All patients were white European older than 18 years and were attended at the Hospital Clínico Universitario of Valladolid (Spain) between April 2008 and November 2012.

The study was carried out according to the Declaration of Helsinki and patients gave their informed consent for being enrolled in the study. This study was approved by the Ethics Committee of Hospital Clínico Universitario (Valladolid) and Instituto de Salud Carlos III (Majadahonda).

### Control groups

We included a control group of 257 patients who underwent major cardiac or abdominal surgery and only developed SIRS^37^. Control group had similar age and gender compared to patients group. They were attended at the same hospital between 2008 and 2012.

Besides, allelic and genotypic frequencies of *CD14* rs2569190 for healthy subjects were collected from the 1000 Genomes Project website (http://www.1000genomes.org/home) in order to compare these frequencies to those obtained from our study. This database covers a wide number of common human genetic variation detected by next generation sequencing in multiple populations^21^. We select the IBS population which involved 107 individuals.

Control group and IBS population were used to compare the allelic and genotypic frequencies of *CD14* rs2569190 with those obtained from our study.

### Clinical data

Major surgery was called to a surgical procedure in which the patient was under general anesthesia and respiratory assistance due to the patient was not able to breathe independently. Epidemiological and clinical data were collected from medical records: age, gender, type of surgery, prior comorbidities such as diabetes, hypertension, chronic kidney disease, chronic obstructive pulmonary disease, liver disease, cardiomyopathy and cancer. In all cardiac surgeries, cardiopulmonary bypass was performed. Severity of sepsis was evaluated by using two ICU scoring systems calculated within the first 24 hours after diagnosis: Acute Physiology and Chronic health Evaluation (APACHE II score)^38^ and Sequential Organ Failure Assessment (SOFA score)^39^.

The septic shock diagnosis was carried out by using the criteria established by the SCCM/ESICM/ACCP/ATS/SIS International Sepsis Definitions Conference^37^. According to clinical findings, infection could be either documented or presumed. In those cases where the presence of infection was strongly suspected without microbiological confirmation, two clinicians with a large experience discussed and reached a consensus diagnosis based on clinical and laboratory results.

### DNA genotyping

Genomic DNA was extracted from whole blood using High Pure PCR Template Preparation kit (Roche Diagnostics GmbH, Mannheim, Germany). *CD14* rs2569190 polymorphism was genotyped at the Spanish National Genotyping Center (CeGen; http://www.cegen.org/). Genotyping was performed by Agena Bioscience’s MassARRAY platform (San Diego, CA, USA) using the iPLEX® Gold assay design system. Duplicated samples were included on each plate to check for technical replicates and negative and positive controls were used on each batch to exclude DNA contamination and ensure a technically correct laboratory process.

### Outcome variable

The outcome variable was the death, censuring at different time points after septic shock diagnosis: 28 days (early mortality), 60 days (middle mortality), and 90 days (late mortality).

### Statistical analysis

Nonparametric tests were used for the description of the study population: Mann-Whitney U test was conducted for continuous variable and chi-squared/Fisher’s exact test for categorical variables. For the genetic association study, Kaplan-Meier and Cox regression analyses were performed to compare the mortality during the first 28, 60 and 90 days according to the presence of *CD14* rs2569190 GG genotype. Statistical analysis was carried out in accordance with dominant, recessive and additive models. These analyses were assessed according to the goodness of fit evaluated by Akaike information criterion (AIC) value and Bayesian information criterion (BIC).

Follow-up was censored at different time points: 28, 60 and 90 days. The Kaplan-Meier product-limit method at 28, 60, and 90 days and Log rank test were used to estimate and compare survival probabilities. Cox regression analyses were used to study the association between *CD14* rs2569190 polymorphism and the mortality during the first 28, 60, and 90 days after diagnosis of septic shock. Each Cox regression was adjusted by the most important clinical and epidemiological co-variables, allowing to avoid the over-fitting of the regression. *CD14* rs2569190 polymorphism was included by forced entry (Enter algorithm) and the most significant co-variables were selected by stepwise algorithm (at each step, co-variables are considered for entry or removal: a p-value for entry and exit of 0.15 and 0.20, respectively). The co-variables used were age, gender, type of surgery, (cardiac or abdominal), elective surgery (emergency or scheduled), comorbidities (obesity, diabetes, hypertension, heart disease, chronic obstructive pulmonary disease (COPD)), liver disease and neoplasia), smoker, and high alcohol intake, antibiotic treatment, peritonitis and APACHE-II score. All statistical analyses were carried out by using the IBM SPSS Statistics for Windows, Version 21.0 (IBM Corp, Chicago, Armonk, NY, USA). All p-values were two-tailed and statistical significance was defined as p < 0.05. Moreover, HWE analyses was performed by Haploview 4.2 software, considering equilibrium when p > 0.05.

### Data availability

All data generated or analyzed during this study are included in this published article. The datasets generated during and/or analyzed during the study are available from the corresponding author on reasonable request.
